# Genetic homogeneity, lack of larvae recruitment, and clonality in absence of females across western Mediterranean populations of the starfish *Coscinasterias tenuispina*

**DOI:** 10.1038/s41598-021-96331-6

**Published:** 2021-08-19

**Authors:** Rocío Pérez-Portela, Alex Garcia-Cisneros, Marta Campos-Canet, Creu Palacín

**Affiliations:** 1 Department of Evolutionary Biology, Ecology and Environmental Sciences, University of Barcelona, 643 Diagonal Avenue, 08028 Barcelona, Spain; 2 Research Institute of Biodiversity (IRBIO), University of Barcelona, Barcelona, Spain

**Keywords:** Biodiversity, Ecological genetics, Molecular ecology, Population genetics, Marine biology

## Abstract

We here analysed the populations’ genetic structure of *Coscinasterias tenuispina,* an Atlantic-Mediterranean fissiparous starfish*,* focusing on the western Mediterranean*,* to investigate: the distribution and prevalence of genetic variants, the relative importance of asexual reproduction, connectivity across the Atlantic-Mediterranean transition, and the potential recent colonisation of the Mediterranean Sea. Individuals from 11 Atlantic-Mediterranean populations of a previous study added to 172 new samples from five new W Mediterranean sites. Individuals were genotyped at 12 microsatellite loci and their gonads histologically analysed for sex determination. Additionally, four populations were genotyped at two-time points. Results demonstrated genetic homogeneity and low clonal richness within the W Mediterranean, due to the dominance of a *superclone*, but large genetic divergence with adjacent areas. The lack of new genotypes recruitment over time, and the absence of females, confirmed that W Mediterranean populations were exclusively maintained by fission and reinforced the idea of its recent colonization. The existence of different environmental conditions among basins and/or density-depend processes could explain this lack of recruitment from distant areas. The positive correlation between clonal richness and heterozygote excess suggests that most genetic diversity is retained within individuals in the form of heterozygosity in clonal populations, which might increase their resilience.

## Introduction

Genetic diversity, needed for populations to adapt to environmental changes, has a profound influence on evolutionary and ecological processes of the species, and is one of the key components of ecosystems biodiversity^1–3^. Understanding how genetic diversity is distributed across populations, and how it arises and is lost, is relevant to estimate species’ resilience under changing conditions and to determine temporal trends of the populations^1,2^.

In marine organisms, spatial distribution of genetic diversity, and so populations’ genetic structure, can largely vary across similar spatial and temporal scales^3–6^ due to the different interplay of biological characteristics (e.g. larva potential dispersal and reproductive strategies) and past and contemporary oceanographic factors (e.g. physical barriers and oceanographic circulation, among others), which generates variable patterns of connectivity^7^.

Marine invertebrates, represented by an abundance of contrasted biological strategies, can have complex reproductive systems that combine sexual and asexual cycles and/or stages. In many benthic invertebrate groups, such as bryozoans, ascidians, or corals, among others, asexual reproduction during adulthood is mostly linked to colonial growing, generating colonies composed of immobile ramets (single individuals generated by clonal propagation)^8^. However, in others, such as some echinoderms, adults and/or larvae can divide into two new free-living individuals, yielding mobile ramets with considerable dispersal capability when the splitting happens at the larval stages^9,10^. In the latter heterogonic animal group, the balance between sexual and asexual reproduction within populations influence the prevalence of ramets and different genetic individuals (genets)^11,12^. This different proportion of ramets and genets can have a strong impact on the intra-population genetic diversity^13^, genetic drift, effective population size, and finally, populations’ divergence and evolutionary potential^14,15^. Theoretical and empirical approaches have suggested that long-term prevalence of asexual reproduction in diploids tends to drive populations to a deviation from the Hardy–Weinberg equilibrium, shifting the fixation coefficient (F_IS_) to negative values^16^, caused by heterozygotic excess due to the accumulation of somatic mutations in absence of recombination^14,15,17^. Additionally, sex-ratio bias in populations dominated by asexual reproduction can also occur if males and females have different energy demands during asexual reproduction^18^.

In echinoderms, asexual reproduction has been reported in three classes: Ophiuroidea, Holothuroidea, and Asterioidea^10^. Within the latter class, we find the starfish genus *Coscinasterias*, with four species described, being all of them fissiparous^10^. In our study, we focus on *Coscinasterias tenuispina* (Lamarck, 1816), a subtropical species widely distributed across both sides of the Atlantic Ocean and the whole Mediterranean Sea^19,20^. Like all representatives of the genus, when *C. tenuispina* sexually reproduces releases a planktonic larva that can live and disperse in the water column for several weeks^21,22^. The most recent genetic analyses, based on both mitochondrial and nuclear markers, demonstrated the existence of two distant evolutionary lineages within *C. tenuispina*: one lineage distributed along the north Atlantic and Mediterranean Sea (hereafter named as *C. tenuispina*); and the south-western Atlantic lineage found along the coast of Brazil (hereafter named as *C. tenuispina* like)^13^. Population genetic analyses also highlighted the importance of asexual reproduction in *C. tenuispina* across the Cantabrian Sea and a some sites at the western Mediterranean, where monoclonal populations were found^13,23^, and also in *C. tenuispina* like along the coast of Brazil where a significant sex-ratio bias was observed^19^. The same studies suggested that the prevalence of asexual propagation is more frequent at the distribution edges of the species^13,23^, and likely related to the constrictions imposed by environmental factors. The temperature was also suggested as the most relevant physical factor correlated to asexual reproduction due to its impact during larvae development in tropical and sub-tropical echinoderms^23,24^. Additionally, significant genetic divergence in *C. tenuispina* among the northeast Atlantic, W Mediterranean, and east Mediterranean basins was detected. The same data suggested a relatively recent colonisation of the Mediterranean Sea from the Atlantic Ocean during one of the last interglacial periods of the Pleistocene^23^, but more data are required to confirm this hypothesis, including the role of the Atlantic-Mediterranean transition in the connectivity patterns between both basins*.*

The role of oceanographic currents across the Gibraltar Strait and the Almeria-Oran front, which delimit the Alboran Sea, on genetic patterns has been explored in species with contrasting biological features, and a differential barrier effect among species demonstrated, although still poorly known in facultative asexual invertebrates^25,26^. Whereas for some species the oceanographic circulation system generates a strong genetic break due to a gene flow interruption^27,28^, for others, a smooth genetic transition (e.g.^29,30^), or even a null effect, was detected due to moderate levels of connectivity between basins (e.g.^31,32^). Additionally, the genetic structure of populations may not be stable over time and can rapidly vary over shorts periods of only a few years (see^30,33^); therefore considering temporal variability in genetic structure studies is desirable to better understand the potential plasticity of populations and to obtain more accurate estimates of gene flow.

In general, studies on *C. tenuispina* have shown unusual biological features in this predator species^20^. Although asexual reproduction can happen all over the year^23^, populations can preserve their potential for sexual reproduction, even when they have not sexually reproduced for long-time periods^23^. Another work performed by Garcia-Cisneros and coauthors^34^, in which telomere length of individuals from wild populations were measured, demonstrated that the clonality rate, at the population level, was significantly and positively correlated with telomere length average. Telomeres, which protect the extreme of the chromosome from DNA damage, shorten after each cellular cycle of mitosis and are routinely used as molecular markers for assessing aging and fitness^35,36^. Hence, higher fitness would be expected when telomeres remain longer during lifetime. The novel finding in *C. tenuispina* suggested that this starfish has a mechanism for telomere elongation during adulthood, never observed in wild animals before, associated with asexual reproduction, and likely related to overcome some of the negative effects related to prolonged periods of asexual reproduction, such as senescence (e.g.^37^).

As presented above, *C. tenuispina* seems to have a large biological and evolutionary plasticity. Based on its reproductive plasticity, and the long dispersal potential of its larva, some authors suggested that *C. tenuispina* could be an efficient colonizer of new areas^9^. Nevertheless, on the other hand, some partially clonal species, from different ecosystems, have suffered large demographic fluctuations and important geographic drifts. In this sense, genetics studies can be applied to indirectly identify major drivers and life history traits associated with populations’ fluctuations^16^ (and references herein). In *C. tenuispina*, there is still an important gap of knowledge related to the levels of genetic diversity and prevalence of clonality across the W Mediterranean area, the effect of the Atlantic-Mediterranean transition on the genetic divergence between basins, and the potential recent colonisation of the Mediterranean basin^34^; genetic features that could determine the long-term resilience of Mediterranean populations of *C. tenuispina*. In order to answer these previous questions, we here explore the populations’ genetic structure of *C. tenuispina*, over space and time, together with information on sex-ratios, focusing on the W Mediterranean area and the Atlantic-Mediterranean transition. Hence, our specific objectives for this study were:

 (1) to investigate levels of genetic diversity, clonality, and structure of W Mediterranean populations of *C. tenuispina*. Information obtained can help to unveil whether current larvae recruitment at some W Mediterranean populations occurs either from adjacent or distant geographical areas, as well as to estimate historical gene flow across the Alboran Sea. We will also determine the distribution limits and prevalence of the different genetic variants found. In *Coscinasterias,* dispersion mainly relies on larvae generated by sexual reproduction. Adults lack migratory behaviour and displacements during adulthood are limited^39^. Therefore, our initial hypothesis is that spatial spreading of clonal lineages by fission would be limited to short geographical distances of only a few kilometres and between close populations.

(2) To explore whether *C. tenuispina* fits within the theoretical models and empirical studies performed in other marine asexual species that relate significant HWE disequilibrium and increasingly deviation of F_IS_ towards negative values, due to heterozygote excess, with modest and high clonality rates (see^16^ and references herein). Our hypothesis is that if those models also apply to *C. tenuispina*, we should observe a positive relationship between clonal diversity and F_IS_ in the populations here analysed. This point is interesting because some authors consider that the heterozygote excess in clonal populations can be an evolutionary advantage to retain genetic diversity within individuals^38^, increasing the potential resilience of populations.

The novelty of our study is combining both spatial and temporal genetic data, together with sex-ratio information, from distant and close populations, within the W Mediterranean, to measure clonality rates and genetic diversity (at the inter- and intra-population, and intra-individual levels). Genetic analyses of populations at two different years allow us to empirically measure the recruitment of new genotypes, and compare it with historical connectivity inferred from coalescence analyses. With all information retrieved, we will finally be able to determine whether Mediterranean populations of *C. tenuispina* have an Atlantic origin from a relatively recent colonisation event. Information generated will contribute to better understand the poorly known genetic patterns of clonal marine invertebrates.

## Results

### Sex determination from gonads

To explore the potential of W Mediterranean populations of *C. tenuispina* for sexual reproduction, we investigated their sex-ratios. Over the 316 individuals of *C. tenuispina* analysed from five W Mediterranean localities (HER, ESC, ALI-14, PAL-14, and LLA), only 60 individuals from two localities, LLA and PAL, had developed gonads (see Supplementary material S1). Histological analysis revealed that all gonads found were testes. Therefore, all sampled individuals with gonads from the W Mediterranean were males, with no females detected at any locality.

### Genetic diversity and clonality

Results of expected and observed heterozygosity (He and Ho, respectively), number of alleles, fixation index (F_IS_), Hardy Weinberg Equilibrium (HWE), number of multi locus genotypes (MLGs) and multi locus lineages (MLLs), and clonal richness from all eight W Mediterranean locations, including all individuals, are presented in Table 1. Information from the whole spatial dataset from different geographical areas (NE Atlantic, W Mediterranean, and E Mediterranean) is included as Supplementary material S2. Our results for the W Mediterranean localities showed larger values of observed than expected heterozygosis under equilibrium. All W Mediterranean localities significantly deviated from the HWE (*p*-value < 0.01) with negative F_IS_ values due to an excess of heterozygotes, when all individuals were included (Table 1). This pattern is also observed when only unique MLGs were considered, although in this case, some sampling sites did not significantly deviated from the HWE (see Supplementary material S2). Hence, although most populations from all areas across the distribution range of the species (NE Atlantic, W Mediterranean, and E Mediterranean) had higher values of Ho than He, and negative F_IS_, this difference was more evident at the W Mediterranean (see and Supplementary material S2 and S3).

**Table 1:** Information of W Mediterranean localities of *C. tenuispina* used for spatial and temporal analyses: location, code, year of collection, number of individuals genotyped (Ng), number of alleles (nA), expected and observed heterozygosity (He and Ho, respectively), F_IS_ considering all individuals (* when *p*-value < 0.01 for HWE), number of MLGs (N_MLG_), number of private MLG (P_MLG_), number of MLLs (N_MLL_), number of private MLL (P_MLL_), and clonal richness (Eff-MLG). For further details see Supplementary material S1 and S2.

Location	Code	Year	Ng	nA	He	Ho	F_IS_	N_MLG_	P_MLG_	N_MLL_	P_MLL_	Eff-MLG
La Herradura	HER (‡)	2014	23	21	0.22	0.35	− 0.67*	10	10	2	2	4.52
Los Escullos	ESC (‡)	2014	20	19	0.24	0.44	− 0.86*	3	3	2	2	1.80
Alicante	ALI-10	2010 (t1)	24	17	0.18	0.34	− 0.95*	2	1	1	0	1.28
	ALI-14 (‡)	2014 (t2)	12	16	0.17	0.33	− 1.0*	1	0	1	0	1.00
Cunit	CUN-11 (‡)	2011 (t1)	24	16	0.17	0.33	− 1.0*	1	0	1	0	1.00
	CUN-14 (‡)	2014 (t2)	–	–	–	–	–	–	–	–	–	–
Palamós	PAL-11 (‡)	2011 (t1)	26	16	0.17	0.33	− 1.0*	1	0	1	0	1.00
	PAL-14 (‡)	2014 (t2)	24	16	0.17	0.33	− 1.0*	1	0	1	0	1.00
Cadaqués	CAI (‡)	2011	23	18	0.18	0.33	− 0.92*	5	4	1	0	1.45
Llançà	LLA-11	2011 (t1)	25	20	0.19	0.33	− 0.82*	3	2	2	1	1.18
	LLA-14 (‡)	2014 (t2)	20	17	0.17	0.33	− 0.90*	2	1	2	1	1.10
Napols	NAP	2013	18	18	0.18	0.33	− 0.87*	2	1	2	1	1.36
Total			239					22	21	7	6	

(‡) New samples collected for this study.

**Figure 1:**
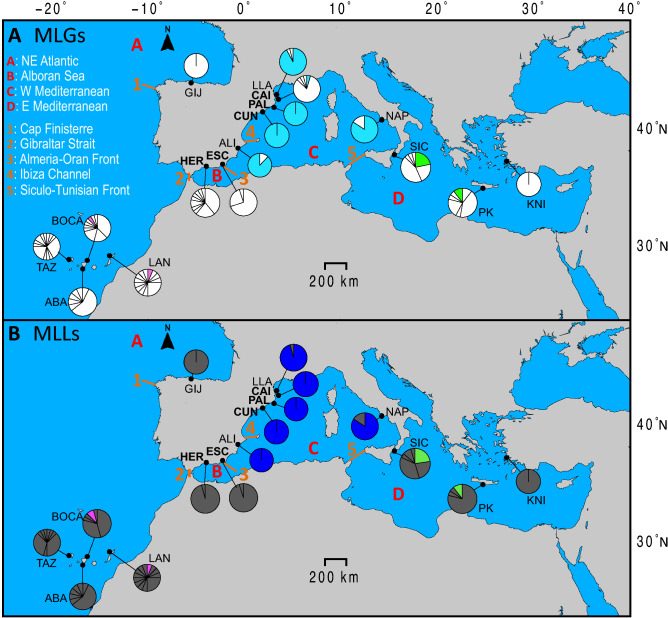
Sampling localities and genetic variants of *Coscinasterias tenuispina*. (**a**) Pie charts depict the frequency of each multi locus genotype (MLG) per locality; shared colours among localities indicate common MLGs, and partitions in white represent private MLGs; (**b**) Pie charts depict the frequency of each multi locus lineage (MLL) per locality; shared colours among localities indicate common MLLs, and partitions in grey represent private MLLs. Major oceanographic areas are indicated by letters (A, B, C, and D) and potential barriers/fronts by numbers (1, 2, 3, 4 and 5). The code of the new sampling sites of this study, located at the Alboran Sea (B) and W Mediterranean (C), are bolded. This map was created with the free software MAPTOOL: SEATURTLE.ORG Maptool. 2002. SEATURTLE.ORG, Inc. http://www.seaturtle.org/maptool/ 06 July 2021.

**Figure 2:**
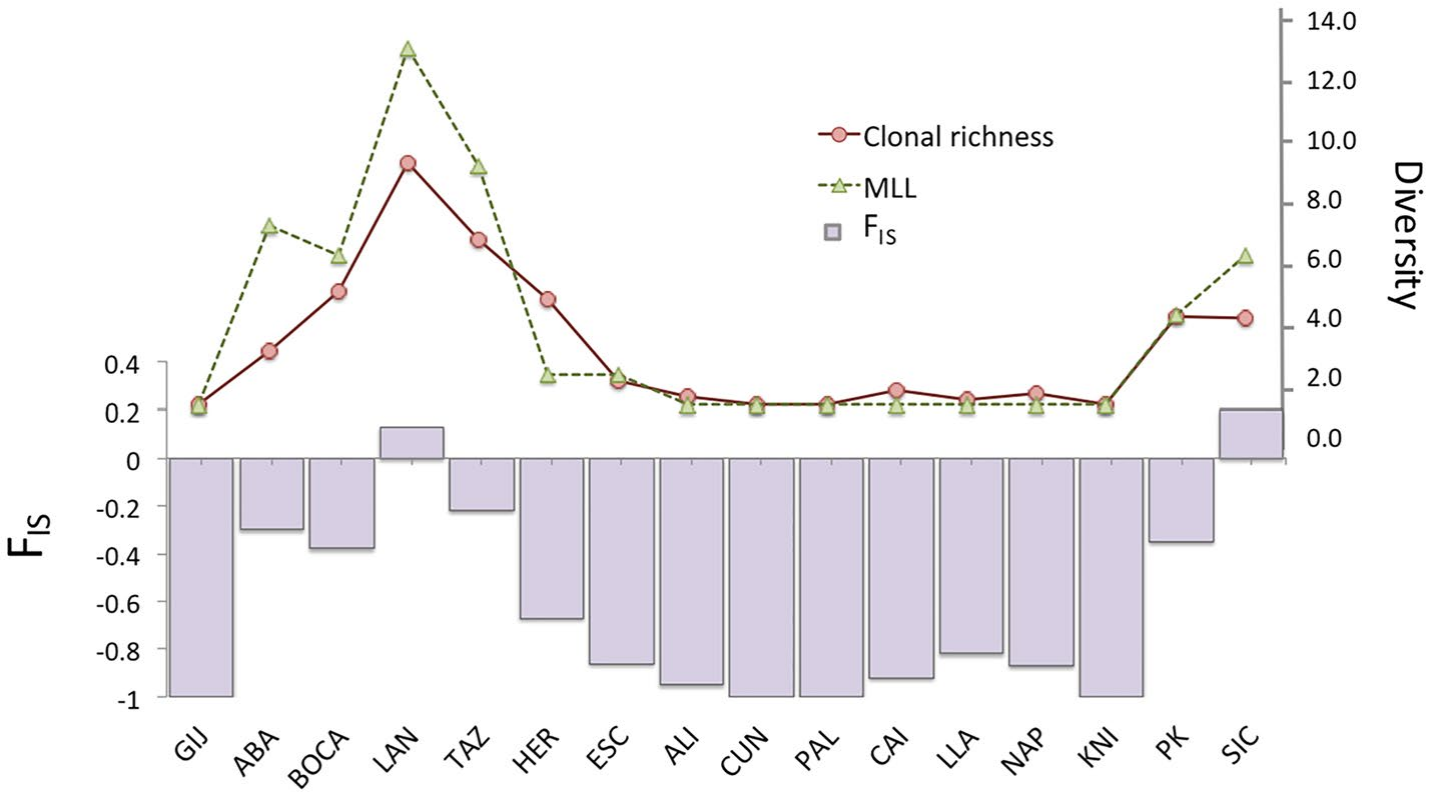
F_IS_ versus genetic diversity in *Coscinasterias tenuispina,* including all individuals. Graph representing the F_IS_ value per locality (left *y*-axis) versus clonal richness and number of MLLs per locality (solid red line and dotted green line, respectively) (right *y*-axis).

For other parameters of genetic diversity such as the number of alleles, number of MGLs and MLLs per locality, and clonal richness, in general, W Mediterranean populations presented lower values for all parameters of diversity, together with the monoclonal populations of KNI (E Mediterranean) and GIJ (Cantabrian Sea, NE Atlantic), compared to NE Atlantic and E Mediterranean populations (see Table 1, Figs. 1 and 2 and Supplementary material S2). Nevertheless, allele richness largely varied between the databases including and excluding individuals sharing the same MLGs (see Supplementary material S4). When all individuals were analysed, allele richness was larger at populations with less incidence of clonality, as in the Canary Islands (NE Atlantic) and E Mediterranean populations, whereas a reverse pattern was obtained when only unique MLGs were considered (see Supplementary material S4). The mean number of MGLs was: 2.4, 4.7, and 11 at the W Mediterranean, E Mediterranean, and Canary Island areas, respectively. The number of MLGs and MLLs, relative to the sample size, per geographical area, was lower in the W Mediterranean (excluding the Alboran Sea) than in the E Mediterranean, the Alboran Sea, and the Canary Islands (see the boxplot graphs at Supplementary material S5). Over the total of 78 MLGs, represented by 52 MLLs, detected across the whole distribution range of *C. tenuispina,* only 22 MLGs and 7 MLLs were identified within the W Mediterranean (including the Alboran Sea), and among them, 10 MLGs were located at the Alboran Sea. It is remarkable the different number of MLGs and MLLs in the W Mediterranean (Fig. 1, Table 1, and Supplementary material S5), including the Alboran Sea. This difference between the number of MLGs and MLLs found at the W Mediterranean, and the Alboran Sea, demonstrates that most of these MLGs are one-allele variants (please see how MLLs were inferred in the Methods section), and suggests the origin of most W Mediterranean MLGs from somatic mutations. Therefore, these MLL are considered to have an asexual origin in absence of recombination. Pie charts on Fig. 1, calculated from MLGs and MLLs frequencies per population, also demonstrate that one MLG dominates most populations of the W Mediterranean area, with the exception of the population of Cadaqués (where this MLG was present but was not the most frequent one), Los Escullos, on the limit of the Almeria-Oran front, and La Herradura at the Alboran Sea. Nevertheless, when we observe the MLLs, all W Mediterranean sites analysed were dominated by one clonal lineage that appeared at high frequency (hereafter named as the *superclone*). Regarding private MLGs and MLLs, a higher frequency of private MLGs and MLLs was observed at the NE Atlantic, Alboran Sea, and E Mediterranean than in the W Mediterranean, being private genotypes at low frequency in the latter area (see Table 1 and Supplementary material S2).

### Relationship between heterozygosity and clonality

All Pearson correlations between the excess of heterozygotes, measured as the difference between expected and observed heterozygosity (He − Ho) and F_IS_, for the dataset including all individuals and that including only unique genotypes, versus genetic diversity descriptors such as number MLLs relative to sample size and clonal richness, were significant. Correlations results of He − Ho versus MLLs and clonal richness were: r = 0.67 and r = 0.65, respectively, all *p* ≤ 0.02 when all individuals were included; and r = 0.64 and r = 0.57, respectively, all *p* ≤ 0.02 when only unique genotypes were included. Correlation results of F_IS_ versus MLLs and clonal richness were: r = 0.83 and r = 0.84, respectively, *p* ≤ 0.00 when all individuals were included; and r = 0,69 and r = 0.57, respectively, *p* ≤ 0.05 when only unique genotypes were included. These results point out a positive correlation between clonal reproduction and heterozygote excess in this species (see some examples of correlation graphs at Supplementary material S6). 

### Genetic structure over space and time

#### Spatial pattern

All population genetic analyses, based on different methods (genetic distances, discriminant analyses of principal components, and Bayesian clustering), showed the W Mediterranean (with eight different localities) as the most homogeneous area across the species’ distribution range.

**Figure 3:**
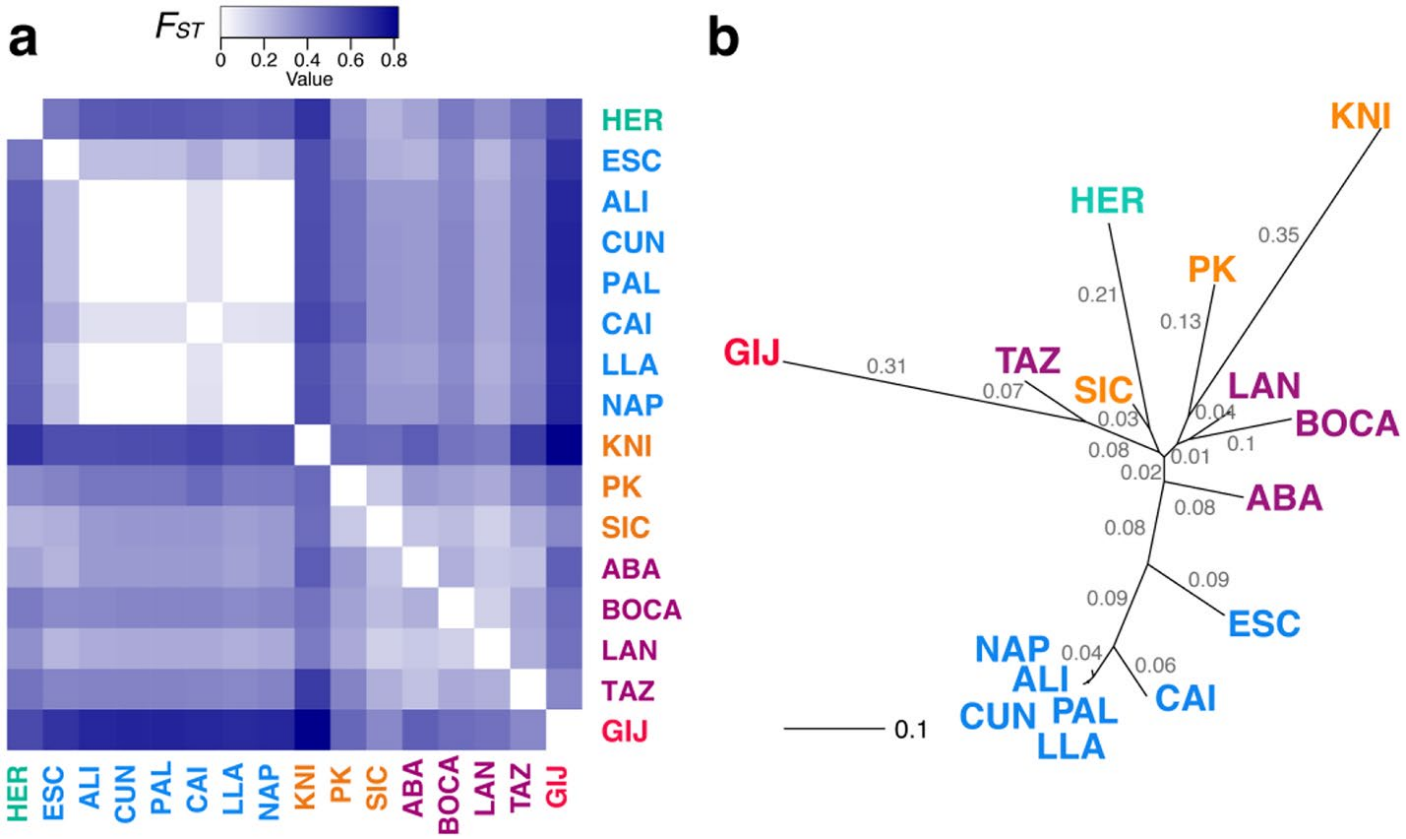
Genetic distances between localities of *C. tenuispina,* including all individuals. (**a**) Heatmap based on *F*_*ST*_ distances between localities and; (**b**) unrooted neighbor-joining tree based on *F*_*ST*_ distances between localities; values on the branches represent genetic distances. Codes in purple represent sites from the Canary Islands, in red from the Cantabrian Sea, turquoise from the Alboran Sea, blue from the W Mediterranean, and orange from the E Mediterranean. On the heatmap, white background comparisons are non-significant (*p* ≤ 0.01). Values of *F*_*ST*_ distances and their associated *p*-values are included as Supplementary material S7. This figure was created with the gplots v 3.0.3 (**a**) and adegenet v 2.1.3 (**b**) packages in R.

Genetic distances between populations, measured with the *F*_*ST*_ and Jost’s *D*_*est*_ tests, displayed similar values of significance between both statistics. When all individuals were included, results were significant (*p* ≤ 0.01) for almost all pairwise comparisons, with the exception of the comparisons between W Mediterranean sites. Within the W Mediterranean, most sites demonstrated low and no significant values of divergence, with the exception of HER (at the Alboran Sea), ESC (at the Alboran Sea limit), and CAI (see values of these statistics at Supplementary material S7). The heatmap displayed in Fig. 3a visually presents the *F*_*ST*_ values calculated from all individuals and localities. The white colour highlights no significant values. The tree constructed from the *F*_*ST*_ matrix suggests the existence of, at least, three main clusters: one of them including W Mediterranean localities (with the exception of HER at the Alboran Sea) and ESC lightly different from the other W Mediterranean sites but still included in this cluster; a second cluster including HER from the Alboran Sea, E Mediterranean populations (although the monoclonal population of KNI was the most distant ones within this cluster) and Atlantic populations from Canary Islands; and the last cluster including the distant population of GIJ, from the Cantabrian Sea (Fig. 3b). When individuals sharing the same MLGs were excluded from the analyses, *F*_*ST*_ values decreased, but divergence patterns among major areas (although most W Mediterranean sites could not be included in these calculations) maintained, with the exception of Sicily that only showed significant genetic differences with HER from the Alboran Sea (see Supplementary material S7), and three populations from the Canary Islands that also lacked genetic differences among them (ABA, BOCA, and LAN).

AMOVA analyses also showed similar results for both databases, either including all individuals or only unique MLGs. In both analyses, significant differences among localities within geographical areas and among geographical areas (NE Atlantic, the Alboran Sea, W Mediterranean, and E Mediterranean) were obtained (F_SC_ = 0.98, *p* = 0.00 and F_CT_ = 4.04, *p* = 0.00 when all individuals included, and F_SC_ = 0.13, *p* = 0.00 and F_CT_ = 0.22, *p* = 0.01 when only unique MLGs were considered).

**Figure 4:**
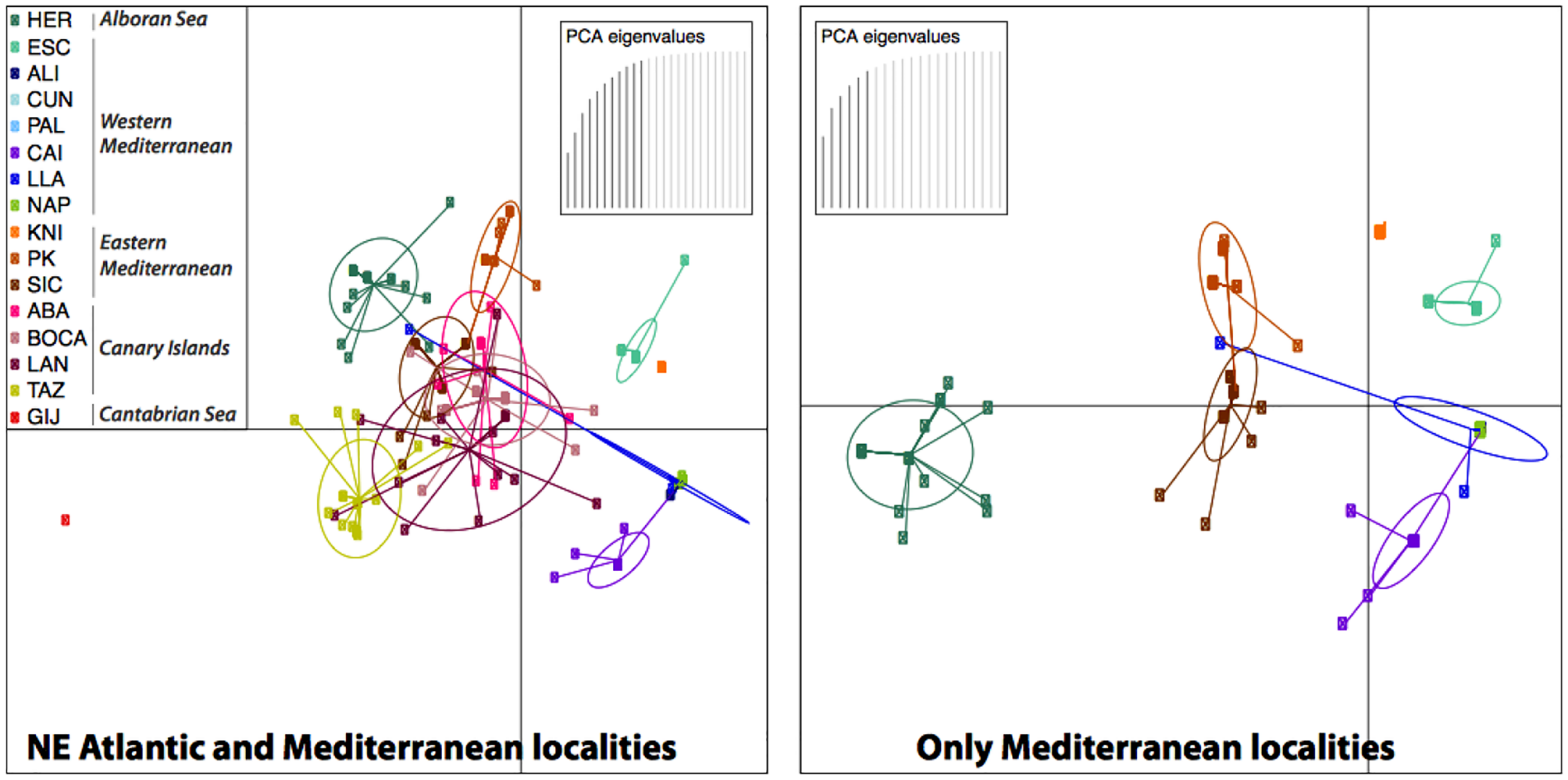
DAPCs of *C. tenuispina*. Graphs depict DAPC results based on sampling localities from two datasets: including all individuals and geographical areas (left graph), and only Mediterranean localities (right graph).

Both DAPC and Structure results evidence coherent patterns with those explained before. The spatial representation of the DAPCs by localities demonstrates a separation between W Mediterranean, E Mediterranean, and Atlantic sites along the horizontal axis, with the overlapping of only one genotype from LLA. Again, DAPCs represent the population at the Alboran Sea limit (ESC) lightly distant to other W Mediterranean localities, and HER, within the Alboran Sea, closely related to the Atlantic pool (see Fig. 4). Interestingly, the E Mediterranean population of KNI seemed to be in the middle between E and W Mediterranean groups, and in some analyses closer to the W Mediterranean than to other E Mediterranean sites. The DAPC performed based on genetic clusters well retrieved genetic differences among the four major marine areas: NE Atlantic, Alboran Sea, W Mediterranean and E Mediterranean (see Supplementary material S8), and showed similar results to those explained below for Structure.

**Figure 5:**
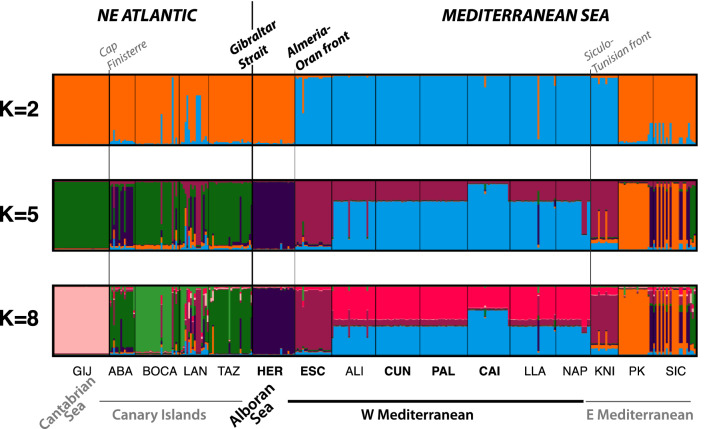
Barplots from the Bayesian clustering analysis obtained in STRUCTURE for K = 2, K = 5 and K = 8, from the whole dataset including all individuals. Major oceanographic areas and fronts are indicated.

The Structure results reveal additional insides on the genetic structure across and within marine areas. The number of K was selected based on the ΔK and maximum likelihood results (Supplementary material S9). The optimal number of genetic clusters (K) were: two, five and eight, and were used for comparison between databases (see Fig. 5 and Supplementary material S9). Results, either including all individuals (Fig. 5) or only unique MLGs per locality (Supplementary material S10), demonstrate the separation of the W Mediterranean populations from all the other genetic pools (for K = 2, K = 5, and K = 8). For K = 5 and K = 8, all individuals of the Alboran Sea (HER) seem to belong to a well-differentiated genetic cluster, and also ESC differentiated to the other W Mediterranean sites. As previously mentioned, it is remarkable the homogeneity of the W Mediterranean area, when considering all individuals, due to the dominance of one clonal lineage. The E Mediterranean population of KNI, again, appeared closely related to the W Mediterranean pool. The population at the Alboran Sea (HER) clustered with the Atlantic and E Mediterranean pools when all individuals were included in the analysis, whereas the opposite pattern, clustering with the W Mediterranean pool occurred when only unique MLGs were considered (see Fig. 5 and Supplementary material S10). Additionally, and despite the large difference in structure between the Alboran Sea population and E and W Mediterranean populations, we observed a few individuals in these two last areas, specifically from SIC and PK, with a high probability to belong to the Alboran Sea cluster (dark purple). This result may suggest minimum gene flow from the Alboran Sea to these two sites at the E Mediterranean.

The relationship among MLGs, based on the minimum spanning networks, was in the line of that explained before (see Supplementary material S11). Most of the W Mediterranean MLGs formed a cluster where all of them were closely related to each other, being divergent by one allele-variant (and therefore the same MLL). MLGs from ESC, at the Alboran Sea limit, were also included in this cluster although genetic distances were larger. There was an Atlantic MLG included in this cluster. All MLGs from the Alboran Sea clustered together and only had distant connections with one W Mediterranean and several Atlantic MLGs. The Atlantic and E Mediterranean MLGs created different clusters that connected with the W Mediterranean MLGs. Results obtained were similar for both databases, including all individuals or unique MLGs per population.

**Figure 6:**
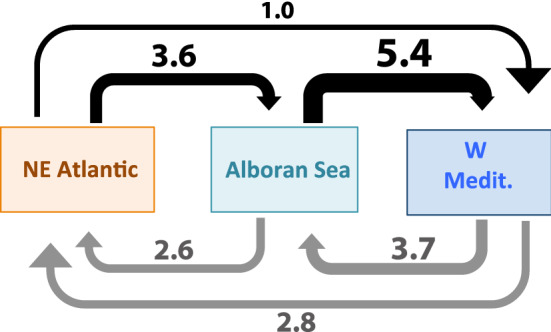
Migration across the Alboran Sea in *C. tenuispina*. Immigration rates estimated with the software Migrate. Gene flow from west to east represented with black arrows, and from east to west with grey arrows.

Migrate results, expressed as the immigration rate between populations (calculated from the M mean), showed limited historical genetic admixture among the NE Atlantic, the Alboran Sea, and the W Mediterranean (see Fig. 6). In general, although immigration rates were relatively low, they suggest higher historical eastward gene flow across the Alboran Sea than in a reverse way. They indicated the important role of the Alboran Sea as a transitional area connecting the Atlantic and Mediterranean basins in the past, with the highest values of genetic interchange found between the Alboran Sea and the W Mediterranean (see specific values in Fig. 6).

#### Temporal trend

**Figure 7:**
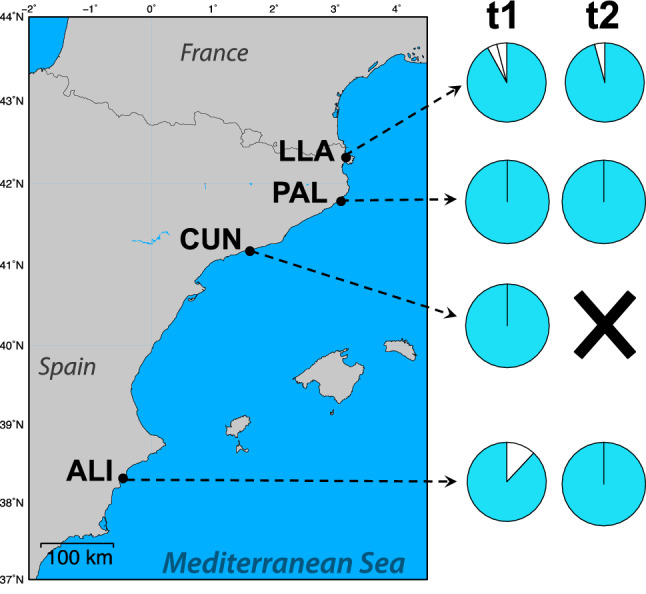
Genetic temporal trend in *C. tenuispina*. Pie charts depict the frequency of each multi locus genotype (MLG) per site at t1 and t2. White partitions represent private MLGs, and blue colour shared genetic variants among localities and over time. For CUN no individuals were found at t2. This map was created with the free software MAPTOOL: SEATURTLE.ORG Maptool. 2002. SEATURTLE.ORG, Inc. http://www.seaturtle.org/maptool/ 23 July 2021.

Neither significant changes in structure (values of F_*ST*_ < 0.01 and *D*_*est*_ = 0.00; and *p*-value > 0.1) or MLG frequencies (see Table 1) were detected in any of the four localities analysed during the temporal monitoring [between time 1 (t1) and time 2 (t2)]. These four localities were already very homogeneous at t1, with all of them dominated by only one genotype (see Fig. 7). Only ALI and LLA had rare genotypes (MLGs) at t1, whose frequencies decreased at t2. For MLLs, there were not differences in frequency between t1 and t2 at any population, with the exception of CUN, a population that disappeared over the monitoring period (Fig. 7). This temporal analysis did not identify the occurrence of new genotypes over time, either from the recruitment of larvae or from somatic mutations.

## Discussion

Our study explores in detail the distribution and frequency of *C. tenuispina* genetic variants within a part of the W Mediterranean sub-basin, over space and time, and includes samples from the Alboran Sea, an important marine transition between the Atlantic Ocean and the Mediterranean Sea (e.g.^25,26^). We also investigate the relationship between heterozygosity and clonality in this fissiparous starfish species.

The general genetic structure of *C. tenuispina* was characterised by large genetic distances between major oceanographic areas, NE Atlantic, W Mediterranean, and E Mediterranean, and across marine fronts, including the Gibraltar Strait, the Almeria-Oran front, and the Siculo-Tunisian front, as demonstrated by genetic distances, Bayesian clustering, and DAPC analyses. Significant differences were also evident between populations within major areas, in concordance with those observed by Garcia-Cisneros and coauthors^13^, with the exception of W Mediterranean localities, the main focus of our current study. Our results demonstrate the existence of a dominant clonal lineage (the *superclone*) widely spread across the W Mediterranean area studied, from Alicante (Spain) to Naples (Italy), covering over 2000 km along the coastline. Although this genetic variant was already detected from three sites by Garcia-Cisneros and coathors^13^, we now demonstrate that the *superclone* is the most frequent clonal lineage across the Mediterranean coast of the Iberian Peninsula and Naples (Italy), being the unique clonal lineage in most localities, but absent in the E Mediterranean, Atlantic Ocean, and Alboran Sea. This clonal dominance generates a pattern of genetic homogeneity within the W Mediterranean area studied that clearly contrasts with those observed from the adjacent areas, the NE Atlantic and E Mediterranean. The strong genetic break between the Alboran Sea (limited by the Gibraltar Strait and the Almeria-Oran front) and the rest of the W Mediterranean, and the genetic homogeneity within the latter area, results from a combination of different biological and genetic features. Among the processes involved in this genetic pattern we can identify: (a) a prevalence of asexual reproduction and clonality within the W Mediterranean area studied, and (b) a lack of contemporary connectivity between the W Mediterranean and adjacent marine areas.

Evidences of clonality, even with populations mostly maintained by asexual rather than sexual reproduction and highly sex-ratio biased^20^, have been found in all species of the genus *Coscinasterias*^11,40–42^, and populations of *C. tenuispina,* and *C. tenuispina* like^13,19,43^. However, clonality, according to the number of shared genetic variants (MLGs and MLLs), clonal richness, heterozygote excess and negative F_IS_ values^16^ (see the Discussion below) was more important in *C. tenuispina* at the W Mediterranean than in any other geographical area and/or species of the genus (e.g.^43^ for *C. tenuispina* like; and^11^ for *C. acutispina*). The presence of the *superclone* in *C. tenuispina* is an extreme case of the clonality, with a unique clonal lineage widely distributed across a large geographical range. Although super-clones has been described in other marine invertebrates, such as corals, their presence was described at smaller spatial scales of only tens of kilometres^44^. In the mentioned cases, the wide expansion and dominance of a clone was attributed to the combination of asexual reproduction together with a well-adapted genotype to local environmental conditions (see^44^ and references herein).

The existence of shared MLGs and low genetic diversity values are genetic signs of asexuality but not exclusive of it^45,46^. The combined information from both parameters together with a deviation from the HWE, negative and significant F_IS_ values and heterozygote excess in *C. tenuispina*^14,15,17,47^, are unequivocal marks of long-term clonal reproduction^13^. Our results demonstrated that *C. tenuispina* fits on the expectations for clonal marine species^16^, with a positive relationship between genetic diversity within populations and the F_IS_ coefficient. Actually, as observed for seagrass species, the correlation between these variables in *C. tenuispina* was weaker when only unique genotypes were considered^16^. Heterozygote excess, due to the accumulation of somatic mutations, was also common in populations of *C. tenuispina* with modest and high rates of asexual reproduction. Hence, the complementary use and interpretation of these genetic variables allowed us identifying clonal reproduction as the origin of the high prevalence of some clones in *C. tenuispina*, instead of inbreeding or methodological problems. The low genetic distances among genetic variants, by only one allele-variant among most low-frequent MLGs, as demonstrated from several analyses, point out their origin in somatic mutations rather than recombination and/or mutations during sexual events.

The genetic pattern and the extreme sex-ratio imbalance, with absence of females, found in the W Mediterranean localities, are in concordance with the biological features previously described for a population of *C. tenuispina*^23^. In a study monitoring the biological cycle of *C. tenuispina* from Llançà (NW Mediterranean), authors found that fission was very frequent all over the year, and although some individuals maintained their potential for gonadal maturation, sexual reproduction was also impeded by the absence of females, among other potential environmental factors such as the low winter temperatures in this area^13,23^. Studies of *C. tenuispina* like along the coast of Brazil also highlighted that some populations were mostly maintained by fission, which drove them to a strong sex-ratio imbalance, and recruitment from larvae negligible^19,48^.

Under conditions of predominant asexual reproduction and absence of contemporary immigration, like those found at the W Mediterranean populations of *C. tenuispina*, genetic divergence among populations is drastically reduced. When strict asexual reproduction is maintained over evolutionary time periods, population size tends to infinite and genetic drift to zero, because genetic diversity cannot be lost in clonal lineages^15,47^, and therefore, homogeneity among populations increases. Our results from the temporal monitoring highlighted that contemporary recruitment of larvae from distant genetic pools is negligible in the W Mediterranean localities. The low levels of connectivity inferred from Migrate across the Alboran Sea likely result from past historical processes and/or shared ancestral polymorphisms rather than current immigration, suggesting a contemporary strong barrier effect to gene flow across this Atlantic-Mediterranean transition. Whereas the potential effect on gene flow across the Siculo-Tunisian Strait has been scarcely investigated in metazoans^49^, but already discussed in *C. tenuispina*^13^, a large body of literature has demonstrated a variety of genetic divergence patterns between Atlantic and Mediterranean populations. Those studied included species with large dispersal potential associated to a planktotrophic larva (see^25,26,30,32,50^, among others), as the one observed in *C. tenuispina*, but patterns inferred from other species contrast with the strong break found in *C. tenuispina*. The genetic patterns and biological information revealed in our study reinforces the idea of a relatively recent colonization of the W Mediterranean basin from the Atlantic during a Pleistocene interglacial period, as already suggested by Garcia-Cisneros and coauthors^13^. During this colonisation, the Alboran Sea might have been functioned as a stepping stone area with higher gene flow eastward, as observed in other species^51^ (and references herein), following the superficial and dominant current circulation across the Gibraltar Strait^52^. From our current data, we cannot determine whether major marine fronts prevent the planktotrophic larvae of *C. tenuispina* to pass across them, or contrary, larvae are able to disperse across marine fronts but recruitment is impeded by other additional factors. Forces impeding larvae and new genotypes recruitment might be varied, such as the existence of the different environmental conditions between basins and/or density-dependent processes after the initial colonisation^53^. The rapid colonisation of the W Mediterranean, potentially marked by a founder effect with a strong reduction of genetic diversity, could have also constrained the dispersal success of additional genotypes due to the exclusion of subsequent dispersers under conditions of high-density^53^.

One question that remains open is how the *superclone* spread all across the W Mediterranean. The absence of sexual reproduction, and therefore, the subsequent spawning, and larvae development, restrict the dispersive potential of the species to the adult stage. Movement rates and migration behaviour are poorly understood in starfish species, but the few studies investigating patterns of adulthood movement in the genus *Coscinasterias* demonstrated that both vertical and horizontal displacement were mainly limited and related to the feeding behaviour^39^. Therefore, with the current data available, we assume that the colonization of the W Mediterranean area by the *superclone* happened by a stepping stone process over an evolutionary time period, and that contemporary migration of individuals is minimum among sites within the W Mediterranean.

In conclusion, *C. tenuispina* presents genetic homogeneity along the W Mediterranean area studied, dominated by a clonal lineage, and genetic divergence with the adjacent marine areas. This genetic pattern may respond to a relatively recent colonization of the W Mediterranean area during an interglacial period, coherent with an ancestral evolutionary gene flow eastward from the Atlantic, and current maintenance of W Mediterranean populations, exclusively, by asexual reproduction after a strong founder effect. In conclusion, our results do not validate our first hypothesis, since clonal lineages seem to be widely spread across broad geographical areas despite the limited dispersal potential of the adult ramets in *Coscinasterias*. On the other hand, despite the low inter- and intra-population diversity along the W Mediterranean area, genetic diversity seems to be retained within individuals, in the form of heterozygosity. Hence, our study confirms that *C. tenuispina* fits with the theoretical models and the empirical data obtained for other facultative asexual marine species. Whether retaining genetic diversity within individuals is evolutionarily advantageous in clonal species is something we cannot conclude from our data. However, if this hypothesis is true, *C. tenuispina* seems to be a resilient species, highly specialised in retaining evolutionary potential, since the species can also elongate its telomeres in absence of sexual reproduction^34^ to avoid processes of senescence.

## Methods

### Sampling and analysis of gonads

#### Spatial sampling

A total of 116 new samples of *C. tenuispina* were collected for spatial analyses at five new localities of the western Mediterranean (W Mediterranean) along the Iberian Peninsula: La Herradura (HER, 36°43′27″N 3°44′12″W), Los Escullos (ESC, 36°48′14″N 2°3′41″W), Cunit (CUN, 41°11′37″N 1°38′50″E), Palamós (PAL, 41°51′17″N 3°08′37″E) and Cadaqués (CAI, 42°17′07″N 3°17′46″E). These new samples were added to the collection already available from another study (see^13^), ranging across > 1200 km of the Iberian Peninsula coast, and Naples (see details in Fig. 1 and Table 1). Two localities were located at the Alboran Sea (HER) and at the Almeria-Oran front limit (ESC). The sampling scheme here presented, together with previous genetic information included five northeast Atlantic and 11 Mediterranean localities (a total of 405 individuals), and passes across five oceanographic fronts and/or transitions: Cap Finisterre, the Gibraltar Strait, the Almeria-Oran front, the Ibiza Channel and the Siculo-Tunisian front (see Fig. 1). Nevertheless, most analyses of this study focus on the W Mediterranean sub-basin, including the Alboran Sea. We collected animals by snorkeling or SCUBA diving in shallow waters (depth ranging between 0 and 5 m), tube feet were removed with forceps from each individual, and preserved in absolute ethanol. Animals that were not used for sex determination (see explanation of “Sex determination from gonads” below) were put immediately back in the sea after tissue collection, and tube feet stored at − 20 °C in ethanol once in the laboratory.

#### Temporal sampling

In order to understand genetic temporal trends in *C. tenuispina*, four geographical sites at the W Mediterranean (LLA, PAL, CUN, and ALI, see codes in Table 1) were sampled at two-time points: time 1 (t1) year 2010 or 2011 depending on the sites, and time 2 (t2) in 2014. The locality of CUN, which was first sampled in 2011 (t1), disappeared during the following years, and none individuals were found or collected during the second sampling survey in 2014. A total of 106 new samples (LLA at t2: LLA-14; PAL at t1 and t2: PAL-11 and PAL-14, respectively; CUN at t1: CUN-11; and ALI at t2: ALI-14) were collected and processes as explained before for the spatial sampling (see Table 1). Data from LLA and ALI at t1 (49 individuals genotyped) were extracted from Garcia-Cisneros and coauthors^13^ to generate a final dataset of 155 individuals for the temporal analyses.

#### Sex determination from gonads

Individuals sampled across the W Mediterranean between the end of October and December in 2014 (see details in Supplementary material S1), when gonads start their maturation^23^, were used for sex determination. For this study, 79 of the new individuals genotyped were also histologically analysed. Animals were collected from four different sampling sites (HER: 23 individuals, ESC: 20 individuals, ALI-14: 12 individuals, and PAL-14: 24 individuals, see Supplementary material S1). Information obtained in this study was added to that from Llança presented in^23^ (237 samples analysed between 2012 and 2014 from Llança) and finally, a total of 316 individuals from five different sites were here included for sex determination (see Supplementary material S1). For other W Mediterranean populations, we did not perform sex determination analysis because animals lacked gonads during the resting period.

For sex determination, animals were collected as explained before, and the whole individuals were preserved in formalin (see^23^). Dissection and visual inspection of all specimens were done to isolate the gonads from all arms. Gonads were then removed from the body and histologically analysed. Briefly, gonads were dehydrated, embedded in paraffin, cut in 5 μm sections using a Microm HM325 Microtome, and stained in haematoxylin–eosin. Sex, male (testis) or female (ovary), was then determined under the optical microscope from the histological preparations. Details are described in^23^.

This study was exempt from ethics approval by the ethics commission of the University of Barcelona. The starfish species studied is not endangered or listed in CITES and its sacrification did not require approval.

### Genetic analyses

#### Genotyping

A previous study on population genetics of *C. tenuispina* demonstrated a high correlation between data from sequences of the mitochondrial gene Cytochrome *c* oxidase I and nuclear microsatellite loci, being the latter more informative for clonality measurements^13^. In the present work, we used microsatellite markers to identify different genotypes, since they better performed for genotypes identification.

For genotyping, we used DNA extracted from tube feet preserved in ethanol at − 20 °C. Total DNA was extracted from 3 to 4 feet using the RedExtract-N-Amp™ Tissue PCR kit, and following the manufacturer’s protocol (Sigma-Aldrich, http://www.sigmaaldrich.com). All specimens were genotyped by twelve polymorphic microsatellites previously described and applied in *C. tenuispina*^13,54^. Microsatellite loci were amplified in multiplex following the protocol described in^13^. Amplification products were analysed on an Applied Biosystems 3730xl DNA Analyzer (Applied Biosystems) at the Scientific-Technical Services of the University of Barcelona. Allele length was estimated relative to the internal GENESCAN 400HD ROX size standard (Applied Biosystems) using the software Peak-Scanner (Thermo Fisher scientific, http://www.thermofisher.com). Allele scoring was graphically performed using the R package MsatAllele^55^. To maintain consistency during the genotyping calling among studies, we used 10 individuals from the previous study^13^ for alleles size calibration. Additionally, the same researchers that scored alleles and genotyped in^13^, scored allele sizes in the present study.

#### Genetic diversity and clonality

From the microsatellite dataset obtained, all individuals per locality and time (t1 and t2 for the temporal analysis) were used to determine the observed and expected heterozygosity (Ho and He, respectively), number of alleles, allele richness, fixation indexes (F_IS_), and the exact test of departure from the Hardy Weinberg Equilibrium (HWE). Genetic descriptors potentially affected by the presence of clones (He, Ho, allele richness, F_IS_ and HWE) were also calculated considering only unique genotypes within the populations. These genetic descriptors were estimated in Genodive v 3.0.4^56^ and R package hierfstat^57^.

Genetic diversity descriptors and clonality levels were explored to infer the prevalence of asexual reproduction in the populations. We applied different statistics previously used in other studies for comparison^13^ (and references herein). We performed in Genodive the following descriptors, for both, the spatial and temporal analyses: number of multi locus genotypes (MLGs), number of multi locus lineages (MLL) and clonal richness (Eff-MLG).

The different MLGs were determined using a distance matrix based on an Infinite Allele Model (IAM) for the whole dataset and locality. Missing data, which only represented 0.65% (for the spatial data) and 0.11% (for the temporal data) were not considered as genetic differences when performing the distance matrix. Therefore only the available scores were used for the identification of MLGs. To estimate the probability of having the same MLG (*P*_*sex*_) due to independent sexual events were used GENCLONE v 2.0^58^. According to the *p*-values obtained (*p* = 0.00), the probability of a repeated MLG arising through sexual events was found negligible, hence we consider all repeat MLGs as a result of asexual events. We also determine the so-called multi locus lineages (MLLs), which are individuals with different MLGs potentially generated by somatic mutations. We detected them using the same method explained before for the MLGs but applying a threshold of only one potential allele of difference between MLGs. Since there is not a standard threshold to determine MLLs generated by somatic mutations, we conservatively allowed only one mutation.

To explore the relationship between heterozygosity and clonality in *C. tenuispina*, we used microsatellite information of all populations from the spatial study, which includes 16 sites. We analysed the potential correlation between the difference between He and Ho (He − Ho) and the number of MLLs (relative to sample size), and clonal richness per locality. Additionally, since some studies have detected heterozygote excess and large deviation from the equilibrium of the F_IS_ statistic towards negative values under conditions of clonal reproduction^16^, we also explored the correlation between F_IS_ and the number of MLLs, and clonal richness. All correlations were computed with the lattice package in R^59^, and two different databases, one including all individuals per site and another only including unique genotypes.

#### Genetic structure over space and time

##### Spatial pattern

Different approaches were used to investigate spatial patterns of genetic structure and gene flow in *C. tenuispina,* with focus on the W Mediterranean area, and including and excluding individuals sharing MLGs.

Genetic distances between pairs of localities, including all individuals, were measured based on two different methods: the *F*_*ST*_ statistics and Jost’s *D*_*est*_ in Genodive. Significant *p*-values were obtained after 10,000 permutations for both statistics. Genetic dissimilarity values for *F*_*ST*_ and *D*_*est*_ values were graphically represented with heatmaps obtained with the gplots v 3.0.3 package for R^60^. The *F*_*ST*_ genetic distances were also used to construct an unrooted neighbour-joining tree with the adegenet package v 2.1.3 in R^61^. Both statistics, *F*_*ST*_ and Jost’s *D*_*est*_*,* were also calculated including unique MLGs, although values of the statistics could not be performed for monoclonal populations or dominated by only one MLG.

Analyses of the Molecular Variance (AMOVAs) were separately run for two databases, including all individuals or only unique genotypes (MLGs) per locality. We used an allele infinite model, and the significance of the tests was evaluated with 10,000 permutations per run in Genodive. We calculated genetic divergence at three hierarchical levels: individuals within localities, localities within different geographical areas and four geographical areas (NE Atlantic, Alboran Sea, W Mediterranean, and E Mediterranean).

Discriminant analyses of principal components (DAPC) were run for two datasets: one including the NE Atlantic and Mediterranean localities, and another only including western Mediterranean localities. Analyses were performed with adegenet. The optimal number of principal components (PC) was determined by the cross-validation method as implemented in this package, and comparison of a-scores for a set of increasing numbers of PCs and a spline interpolation using the a-score function. The number of genetic clusters that better explain our data was explored with the function find.clusters. DAPC analyses were performed grouping samples by actual localities (sampling sites) and also by the genetic clusters inferred from our data. Representing DAPC by clusters helps to determine how well the selected clusters retrieve the actual populations.

The software STRUCTURE v 2.3.4^62^ was also used to infer an optimal number of homogeneous genetic units (K) based on Bayesian clustering analyses. We run two analyses, one included all individuals and another included only unique MLGs, with K numbers from 1 to 12 in both cases. For each K run, 200,000 Markov chain Monte Carlo (MCMC) steps were performed following 80,000 burn-in iterations. Ten independent replicates per run were computed under the “admixture model” implemented by the software. Convergence of the analyses was confirmed verifying that key statistical parameters (alpha and *F*_ST_) reached the equilibrium. The most likely value of clusters was identified comparing the rate of change in the likelihood of K. The optimal K values were determined using the ad hoc statistic ΔK^63^ and the maximum likelihood. The 10 independent replicates per run were averaged and aligned using the clumpak server^64^ and results graphically represented with the same software.

Additionally to the previous analyses, minimum spanning networks based on dissimilarity (number of different alleles), from both databases, one including all individuals and another including only unique MLGs, were constructed to visualise the relationships among genetic variants (MLGs) from the different geographical areas with the package poppr v 2.9.2^65^ in R.

To explore evolutionary or historical migration patterns between the NE Atlantic and W Mediterranean areas, and across the Alboran Sea, we used the software Migrate v 3.6.11^66^. For migration analyses, we only considered unique MLGs per geographical area (NE Atlantic, Alboran Sea, and W Mediterranean). Due to the computational intensity of analysis in Migrate, only the Alboran Sea and adjacent areas, the main focus of our paper, were included in the analysis. We estimate asymmetrical migration (M) among geographical areas based on Bayesian inference using coalescence. Migration per generation is expressed as 4N*m* in nuclear markers, in which N is the effective population size, also estimated in Migrate, and *m* the immigration rate. Since the mutation model for our microsatellite loci is known, the Brownian motion mutation model, recommended for microsatellites^65^, with a constant mutation rate for all loci was used as implemented in the software. A custom-migration model of recurrent immigration among the three areas in absence of divergence was applied. A first preliminary run of our data was performed to infer the *prior* distribution of parameters and their boundaries. Three additional preliminary runs were also computed increasing the number of sampled steps and the increment between samples. After each run, histograms of the parameters obtained, as well as their *Effective Sample Size* (ESS), were explored to determine whether the length of the run was appropriate. For the definitive run, and considering all the parameters and boundaries obtained from the preliminary runs, we finally performed three different replicates of one long chain, with an increment between the samples (long-inc) of 1,600,000 and the number of sampled steps (long-sample) of 800,000, burning of 480,000, and an adaptive heating scheme of four different temperatures (1.0, 1.5, 3.0 and 1000.0).

##### Temporal trend

The frequency of the MLGs and MLLs found per locality at t1 and t2 was calculated and represented as pie charts. Genetic distances between years, t1 and t2, for all four W Mediterranean sites were measured based on *F*_*ST*_ and Jost’s *D*_*est*_ statistics. Diversity and clonality indexes were also calculated per site and year as explained before.

## Supplementary Information

Supplementary Information.

## Data Availability

The complete dataset of microsatellite genotyping in GenALEx format has been deposited at Mendeley Data (https://doi.org/10.17632/gwxfczs4d4.1).
