# Supplementary material for: Genetic homogeneity, lack of larvae recruitment, and clonality in absence of females across western Mediterranean populations of the starfish Coscinasterias tenuispina
Source: Sci Rep. 2021 Aug 19;11:16819. doi: 10.1038/s41598-021-96331-6 (PMC8376918; doi:10.1038/s41598-021-96331-6)
Supplement: Supplementary file 1 — Supplementary Information. [file 41598_2021_96331_MOESM1_ESM.pdf]

Genetic homogeneity, lack of larvae recruitment, and clonality in absence of females across western Mediterranean populations of the starfish *Coscinasterias tenuispina*

Rocío Pérez-Portela*[1,2], Alex Garcia-Cisneros[1], Marta Campos-Canet[1], Creu Palacín[1,2]

**Location**	**Code**	**Year**	**n**	$\mathbf{n_{Ø}}$	$\mathbf{n_m}$	$\mathbf{n_f}$
La Herradura	HER (‡)	2014	23	23	0	0
Los Escullos	ESC (‡)	2014	20	20	0	0
Alicante	ALI-14 (‡)	2014	12	12	0	0
Palamós	PAL-14 (‡)	2014	24	19	5	0
Llançà	LLA	2012-2014	237	182	55	0
Total			316	256	60	0

Supplementary material S1. Information of W Mediterranean localities of *C. tenuispina* used for histological analyses and sex determination: location, code, year/s of collection, number of individuals histologically analysed (n), number of immature individuals detected ($n_{Ø}$), number of males detected (n_m), and number of females detected (n_f).
(‡) New samples collected for this study.

Code	Area	Ng	nA	He	He(1)	Ho	Ho(1)	N_{MLG}	P_{MLG}	N_{MLL}	P_{MLL}	Eff-MLG	F_{IS}	$F_{IS}(1)$
GIJ	Cantabrian Sea, NE Atlantic	30	14	0.08	0.08	0.17	0.17	1	1	1	1	1.00	-1.00**	-
ABA	NE Atlantic	14	24	0.34	0.38	0.44	0.38	7	7	7	7	2.80	-0.30**	0.00
BOCA	NE Atlantic	24	25	0.41	0.45	0.57	0.52	10	9	6	5	4.80	-0.38**	-0.14*
LAN	NE Atlantic	16	27	0.32	0.35	0.28	0.33	13	12	13	12	9.14	0.13*	0.06
TAZ	NE Atlantic	24	28	0.29	0.32	0.36	0.34	13	13	9	9	6.55	-0.22**	-0.08
HER	Alboran Sea, W Mediterranean	23	21	0.22	0.24	0.36	0.36	10	10	2	2	4.52	-0.67**	-0.47**
ESC	W Mediterranean	20	19	0.24	0.29	0.44	0.44	3	3	2	2	1.80	-0.86**	-0.52**
ALI-10	W Mediterranean	24	17	0.18	0.21	0.34	0.38	2	1	1	0	1.28	-0.95**	-0.80**
CUN-11	W Mediterranean	24	16	0.17	0.17	0.33	0.33	1	0	1	0	1.00	-1.00**	-
PAL-11	W Mediterranean	26	16	0.17	0.17	0.33	0.33	1	0	1	0	1.00	-1.00**	-
CAI	W Mediterranean	23	18	0.18	0.19	0.33	0.32	5	4	1	0	1.45	-0.92**	-0.65**
LLA-11	W Mediterranean	25	20	0.19	0.31	0.33	0.33	3	2	2	1	1.18	-0.82**	-0.09
NAP	W Mediterranean	18	18	0.18	0.21	0.33	0.33	2	1	2	1	1.36	-0.87**	-0.60
KNI	E Mediterranean	15	14	0.09	0.09	0.17	0.17	1	1	1	1	1.00	-1.00**	-
PLK	E Mediterranean	19	25	0.27	0.33	0.36	0.38	7	6	4	3	3.97	-0.35**	-0.17
SIC	E Mediterranean	23	26	0.31	0.37	0.25	0.25	6	5	6	5	3.92	0.20**	0.32**
TOTAL		348						78	75	52	49			

Sumplementary material S2. Genetic descriptors of *C. tenuispina* localities used for spatial analyses, including all geographical areas. Locality/population code, geographical area, number of individuals included for population genetic analyses (Ng), number of alleles (nA), expected and observed heterozygosity (He and Ho, respectively) including all individuals, expected and observed heterozygosity considering only unique genotypes (He(1) and Ho(1), respectively), number of MLGs (N_{MLG}), number of private MLGs (P_{MLG}), number of MLLs (N_{MLL}), number of private MLLs (P_{MLL}), clonal richness (Eff-ML), and F_{IS} considering all individuals and only unique genotypes ($F_{IS}(1)$) (* when *p*-value < 0.01 for HWE).

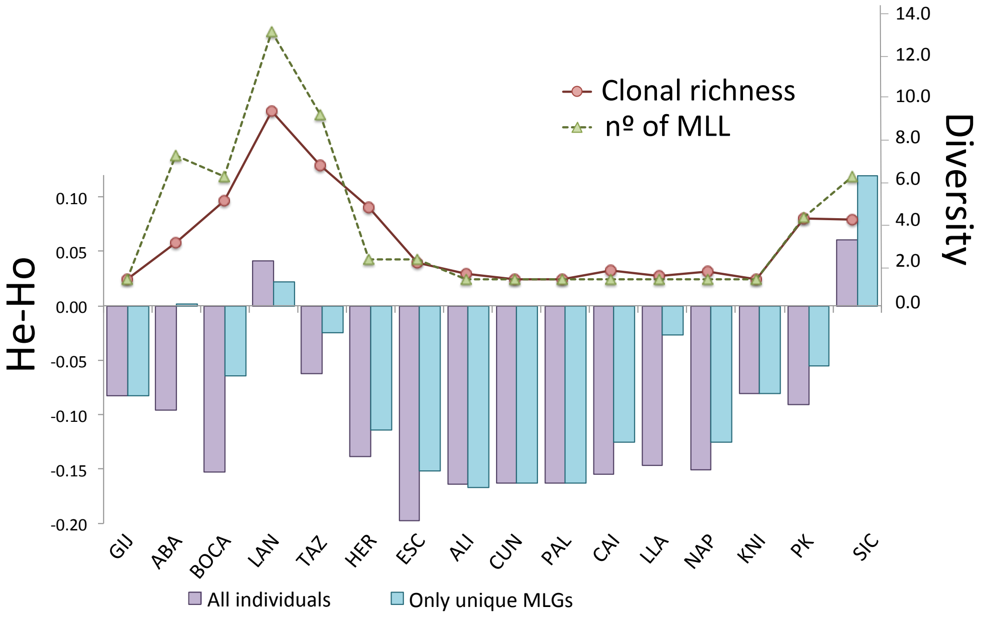

Sumplementary material S3. Heterozygosity *versus* genetic diversity in *Coscinasterias tenuispina*. Graph representing the difference between expected and observed heterozygosity (He-Ho) per locality (left *y*-axis), including all individuals per locality or only unique MLGs (purple and blue bars. respectively) *versus* genetic diversity (right *y*-axis), measured as clonal richness and number of MLLs per locality (solid red line and dotted green line, respectively).

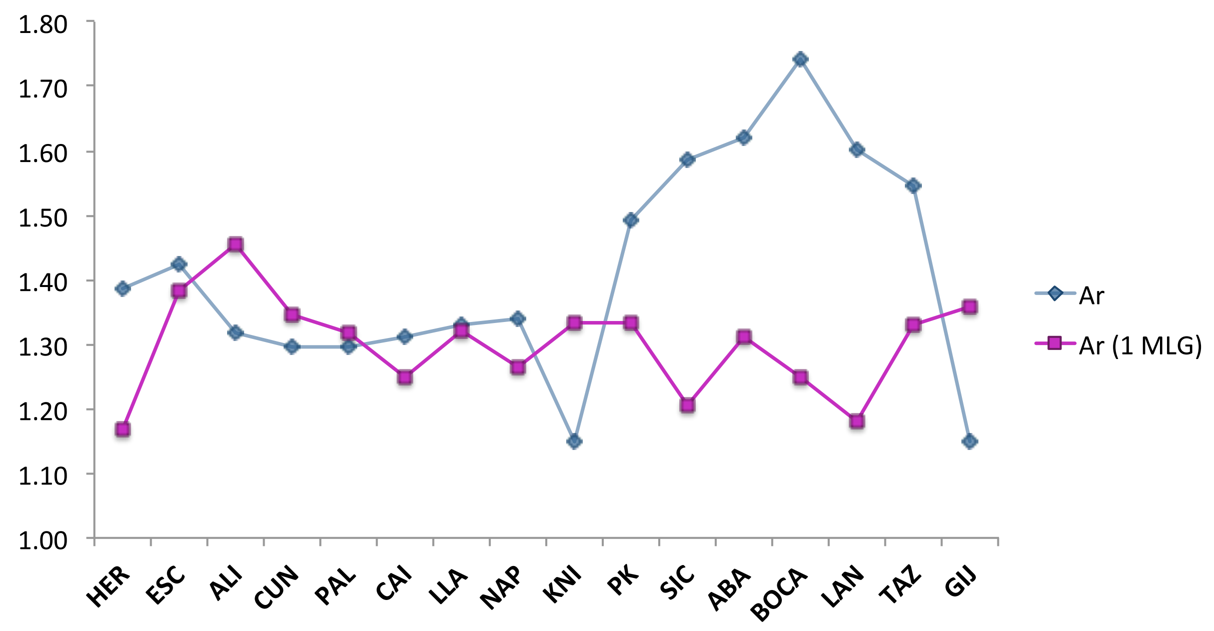

Supplementary material S4. Allele richness (Ar) in *C. tenuispina* localities. Ar: when all individuals were considered, and Ar (1 MLG): allele richness when only unique MLGs were considered.

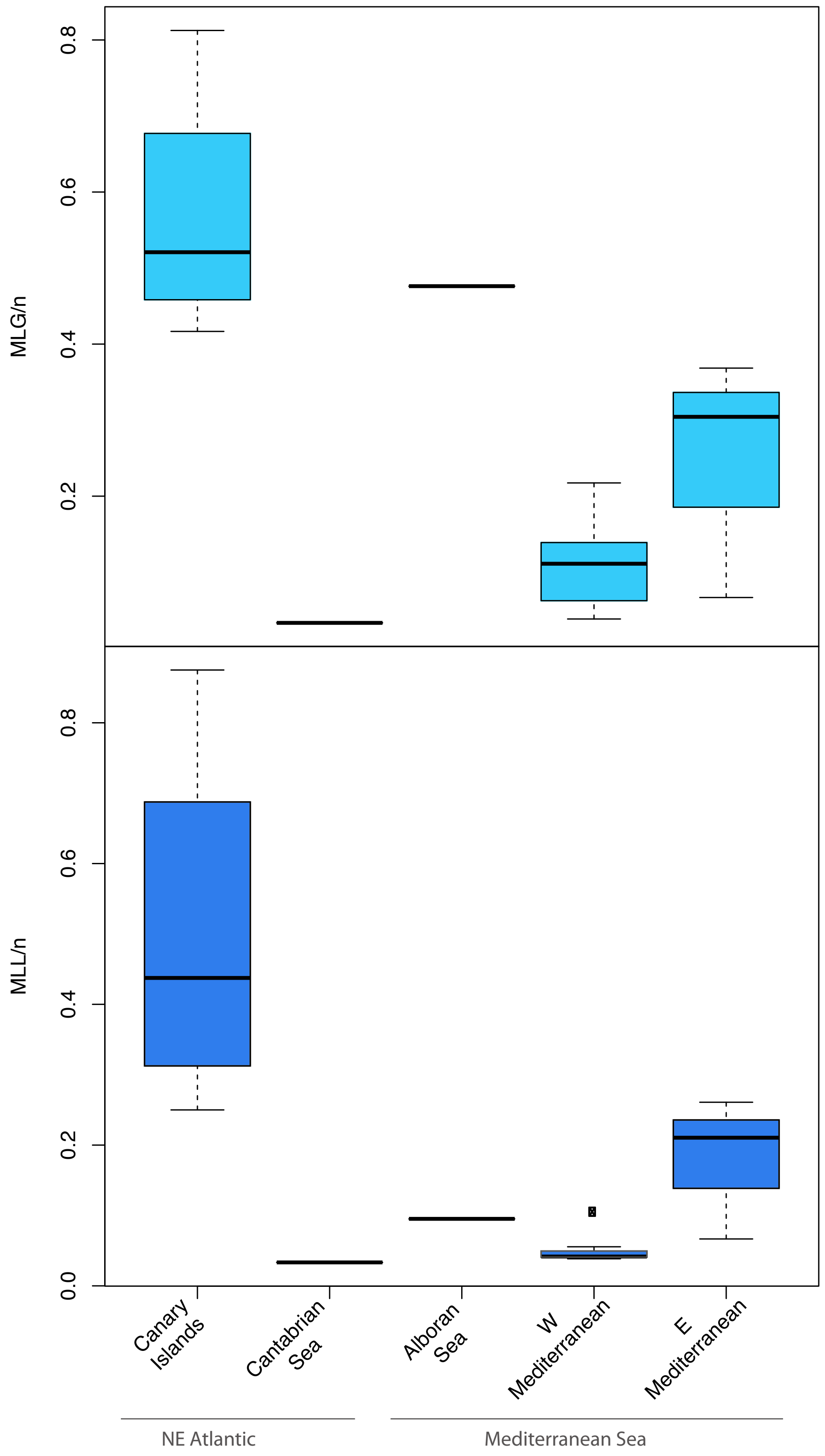

Supplementary material S5 Number of MLGs and MLLs. Boxplot graphs representing the number of MLGs and MLL, relative to sample size (n), per geographical area in *C. tenuispina*.

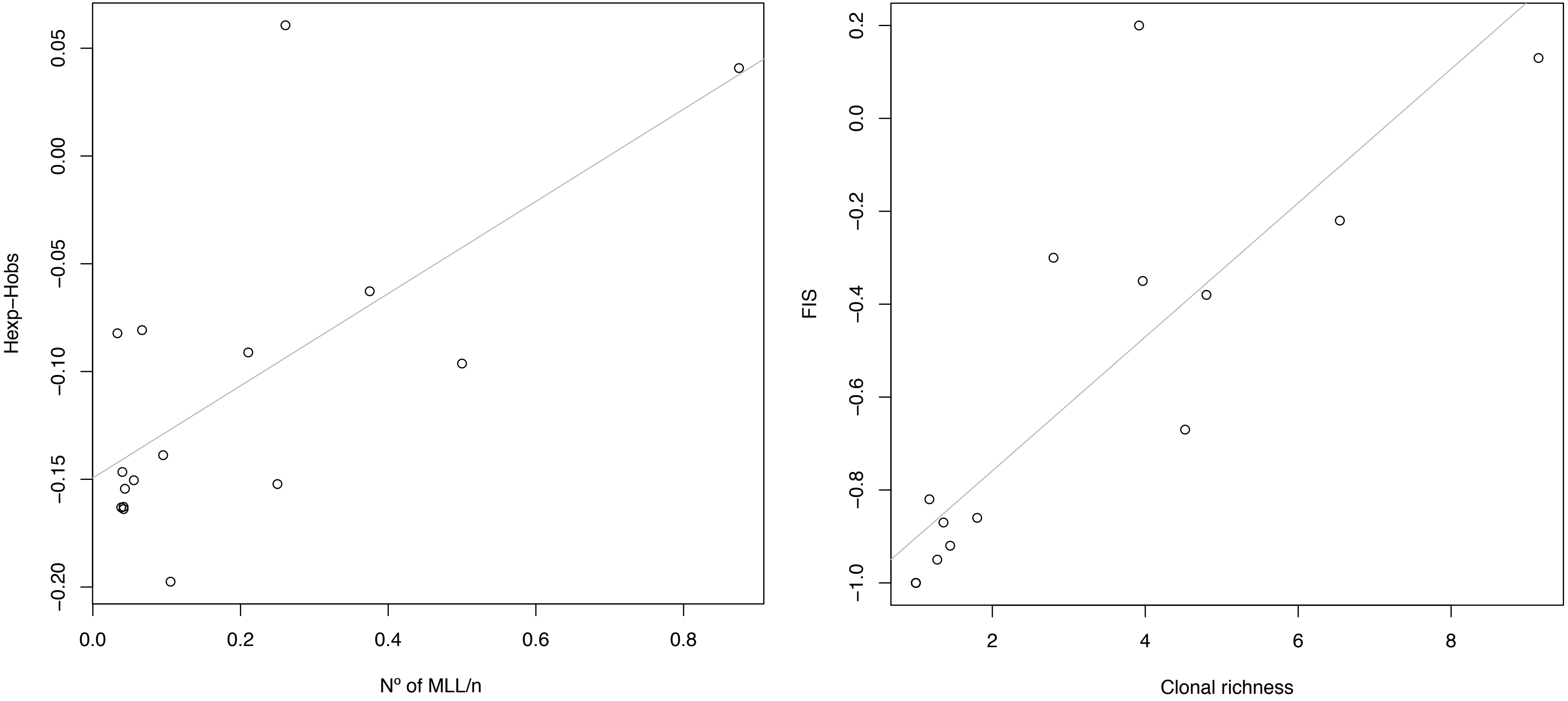

Supplementary material S6. Correlation results including all individuals sampled. On the left: Graphical representation of the correlation between He-Ho (Hexp-Hobs) and number of MLL relative to sample size (Nº of MLL/n) (correlation value: r=0.64; *p*= 0.004). On the right: Graphical representation of the correlation between F_{IS} (FIS) and clonal richness (correlation value: r=0.84; *p*= 0.000).

F_{ST} INCLUDING ALL INDIVIDUALS

	HER	ESC	ALI	CUN	PAL	CAI	LLA	NAP	KNI	PK	SIC	ABA	BOCA	LAN	TAZ	GIJ
**HER**	--	0.00	0.00	0.00	0.00	0.00	0.00	0.00	0.00	0.00	0.00	0.00	0.00	0.00	0.00	0.00
**ESC**	0.44	--	0.00	0.00	0.00	0.00	0.00	0.00	0.00	0.00	0.00	0.00	0.00	0.00	0.00	0.00
**ALI**	0.53	0.21	--	0.23 NS	0.10 NS	0.00	0.11 NS	0.02 NS	0.00	0.00	0.00	0.00	0.00	0.00	0.00	0.00
**CUN**	0.54	0.21	0.00	--	1.00 NS	0.00	1.00 NS	0.08 NS	0.00	0.00	0.00	0.00	0.00	0.00	0.00	0.00
**PAL**	0.54	0.21	0.00	0.00	--	0.00	0.24 NS	0.07 NS	0.00	0.00	0.00	0.00	0.00	0.00	0.00	0.00
**CAI**	0.53	0.26	0.10	0.10	0.10	--	0.00	0.00	0.00	0.00	0.00	0.00	0.00	0.00	0.00	0.00
**LLA**	0.51	0.19	0.00	0.00	0.00	0.09	--	0.07 NS	0.00	0.00	0.00	0.00	0.00	0.00	0.00	0.00
**NAP**	0.53	0.21	0.01	0.00	0.00	0.10	0.00	--	0.00	0.00	0.00	0.00	0.00	0.00	0.00	0.00
**KNI**	0.65	0.56	0.56	0.57	0.57	0.59	0.55	0.56	--	0.00	0.00	0.00	0.00	0.00	0.00	0.00
**PK**	0.37	0.40	0.43	0.44	0.44	0.48	0.42	0.43	0.48	--	0.00	0.00	0.00	0.00	0.00	0.00
**SIC**	0.24	0.26	0.33	0.33	0.33	0.33	0.31	0.33	0.47	0.17	--	0.00	0.00	0.00	0.00	0.00
**ABA**	0.29	0.24	0.32	0.33	0.33	0.32	0.30	0.32	0.52	0.32	0.19	--	0.00	0.00	0.00	0.00
**BOCA**	0.42	0.38	0.38	0.39	0.39	0.39	0.38	0.39	0.45	0.30	0.22	0.26	--	0.00	0.00	0.00
**LAN**	0.35	0.24	0.26	0.28	0.28	0.28	0.26	0.27	0.42	0.27	0.15	0.18	0.15	--	0.00	0.00
**TAZ**	0.44	0.39	0.39	0.40	0.40	0.39	0.38	0.39	0.62	0.40	0.25	0.20	0.28	0.26	--	0.00
**GIJ**	0.58	0.65	0.69	0.70	0.70	0.69	0.68	0.69	0.82	0.49	0.38	0.51	0.47	0.46	0.38	--

F_{ST} INCLUDING ONLY UNIQUE MLGs

	ABA	BOCA	LAN	TAZ	HER	ESC	CAI	PK	SIC
**ABA**	--	0.03 NS	0.01 NS	0.00	0.00	0.02 NS	0.00	0.00	0.07 NS
**BOCA**	0.07	--	0.13 NS	0.00	0.00	0.02 NS	0.00	0.00	0.07 NS
**LAN**	0.08	0.03	--	0.00	0.00	0.00	0.00	0.00	0.13 NS
**TAZ**	0.15	0.15	0.16	--	0.00	0.00	0.00	0.00	0.01 NS
**HER**	0.27	0.27	0.30	0.36	--	0.00	0.00	0.00	0.00
**ESC**	0.10	0.17	0.17	0.31	0.37	--	0.02 NS	0.022 NS	0.11 NS
**CAI**	0.23	0.23	0.20	0.30	0.44	0.22	--	0.00	0.02 NS
**PK**	0.26	0.15	0.20	0.31	0.30	0.27	0.36	--	0.05 NS
**SIC**	0.07	0.06	0.04	0.10	0.24	0.13	0.19	0.11	--

Jost's D*est* INCLUDING ALL INDIVIDUALS

	HER	ESC	ALI	CUN	PAL	CAI	LLA	NAP	KNI	PK	SIC	ABA	BOCA	LAN	TAZ	GIJ
**HER**	--	0.00	0.00	0.00	0.00	0.00	0.00	0.00	0.00	0.00	0.00	0.00	0.00	0.00	0.00	0.00
**ESC**	0.22	--	0.00	0.00	0.00	0.00	0.00	0.00	0.00	0.00	0.00	0.00	0.00	0.00	0.00	0.00
**ALI**	0.27	0.07	--	0.24 NS	0.10 NS	0.00	0.11 NS	0.02 NS	0.00	0.00	0.00	0.00	0.00	0.00	0.00	0.00
**CUN**	0.27	0.07	0.00	--	1.00 NS	0.00	1.00 NS	0.08 NS	0.00	0.00	0.00	0.00	0.00	0.00	0.00	0.00
**PAL**	0.27	0.07	0.00	0.00	--	0.00	0.24 NS	0.07 NS	0.00	0.00	0.00	0.00	0.00	0.00	0.00	0.00
**CAI**	0.27	0.09	0.02	0.02	0.02	--	0.00	0.00	0.00	0.00	0.00	0.00	0.00	0.00	0.00	0.00
**LLA**	0.26	0.06	0.00	0.00	0.00	0.02	--	0.07 NS	0.00	0.00	0.00	0.00	0.00	0.00	0.00	0.00
**NAP**	0.27	0.07	0.00	0.00	0.00	0.02	0.00	--	0.00	0.00	0.00	0.00	0.00	0.00	0.00	0.00
**KNI**	0.33	0.24	0.19	0.19	0.19	0.21	0.19	0.19	--	0.00	0.00	0.00	0.00	0.00	0.00	0.00
**PK**	0.19	0.22	0.22	0.22	0.22	0.26	0.21	0.22	0.20	--	0.00	0.00	0.00	0.00	0.00	0.00
**SIC**	0.11	0.13	0.16	0.16	0.16	0.16	0.15	0.16	0.22	0.09	--	0.00	0.00	0.00	0.00	0.00
**ABA**	0.16	0.13	0.17	0.16	0.16	0.16	0.15	0.17	0.30	0.21	0.12	--	0.00	0.00	0.00	0.00
**BOCA**	0.33	0.29	0.25	0.26	0.26	0.26	0.25	0.27	0.27	0.22	0.16	0.21	--	0.00	0.00	0.00
**LAN**	0.20	0.12	0.12	0.12	0.12	0.12	0.12	0.12	0.18	0.16	0.08	0.11	0.11	--	0.00	0.00
**TAZ**	0.27	0.23	0.20	0.20	0.20	0.19	0.19	0.20	0.39	0.26	0.15	0.12	0.21	0.15	--	0.00
**GIJ**	0.24	0.35	0.34	0.33	0.33	0.33	0.33	0.34	0.41	0.20	0.15	0.28	0.29	0.21	0.14	--

Jost's D*est* INCLUDING ONLY UNIQUE MLGs

	ABA	BOCA	LAN	TAZ	HER	ESC	CAI	PK	SIC
**ABA**	--	0.03 NS	0.01 NS	0.00	0.00	0.04 NS	0.00	0.00	0.07 NS
**BOCA**	0.06	--	0.13 NS	0.00	0.00	0.02 NS	0.00	0.00	0.08 NS
**LAN**	0.05	0.02	--	0.00	0.00	0.00	0.00	0.00	0.14 NS
**TAZ**	0.09	0.10	0.10	--	0.00	0.00	0.00	0.00	0.01 NS
**HER**	0.16	0.20	0.18	0.23	--	0.00	0.00	0.00	0.00
**ESC**	0.07	0.15	0.12	0.21	0.21	--	0.02 NS	0.02 NS	0.08 NS
**CAI**	0.13	0.17	0.11	0.17	0.22	0.08	--	0.00	0.02 NS
**PK**	0.19	0.12	0.13	0.21	0.17	0.18	0.21	--	0.05 NS
**SIC**	0.04	0.05	0.02	0.05	0.12	0.09	0.10	0.06	--

Supplementary material S7. Genetic distances between localities of *C. tenuispina*. Values of the F_{ST} and Jost's D*est* for databases including all individuals or only unique MLGs. The value of the statistic is represented below the diagonal, and the associated *p*-value above the diagonal. NS: Non-significant after false discovery rate (FDR) correction[1] set at $p \leq 0.01$ for both datasets.

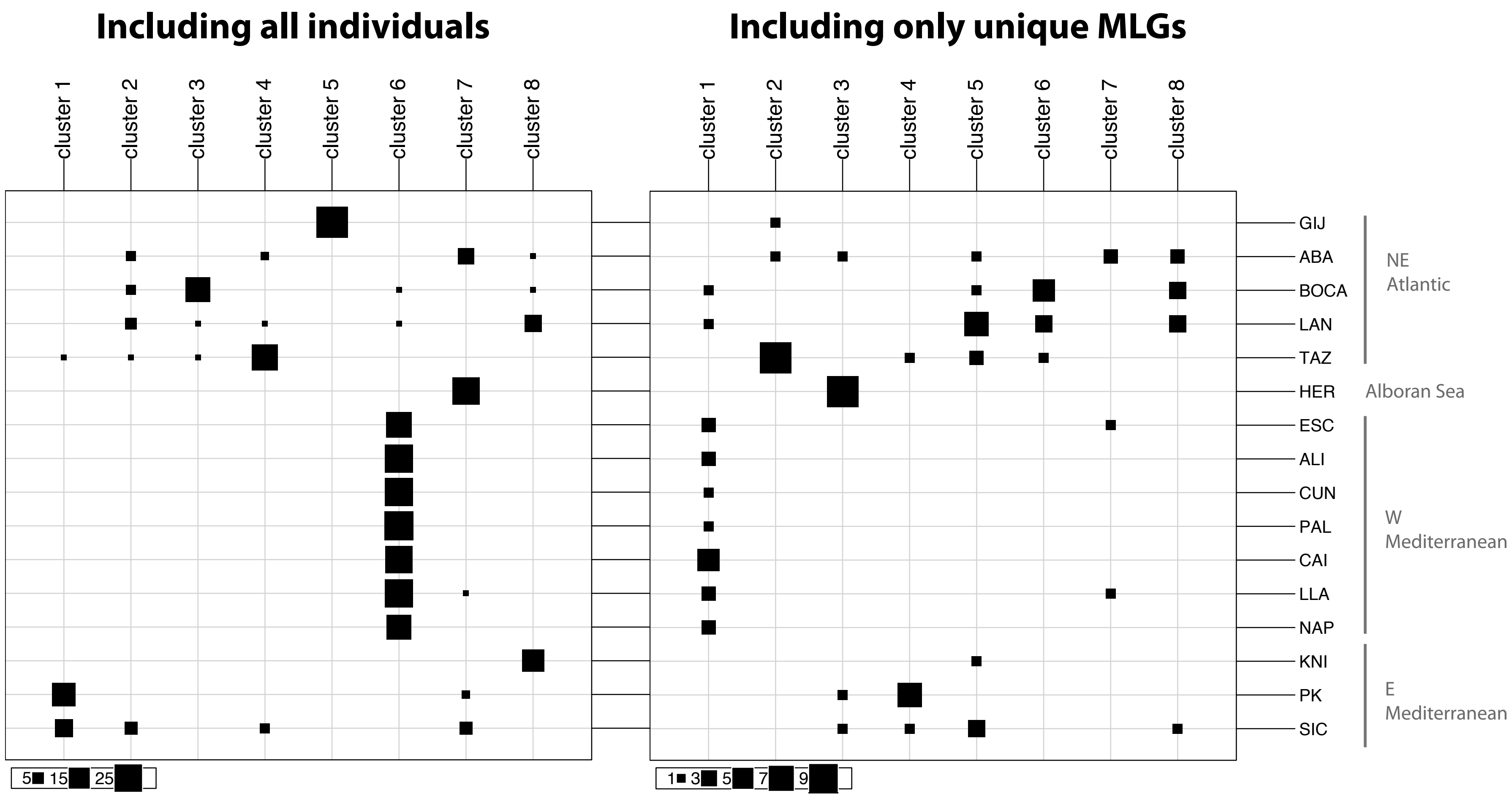

Supplementary material S8. The contingency tables of inferred genetic clusters (K=8) *versus* actual localities from DAPC (8 clusters were selected for comparison with Structure results). Columns correspond to inferred genetic clusters and rows to sampling localities. Tables have been performed from two datasets, one including all individuals and another including only unique MLGs.

Including all individuals

ΔK

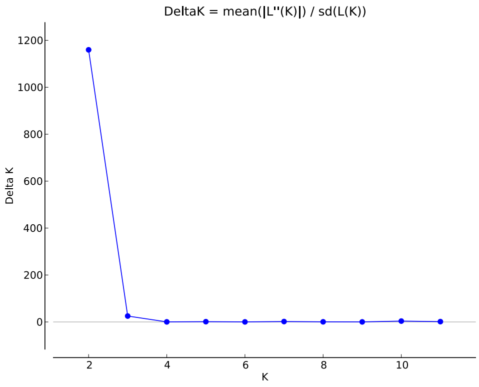

Maximum Likelihood

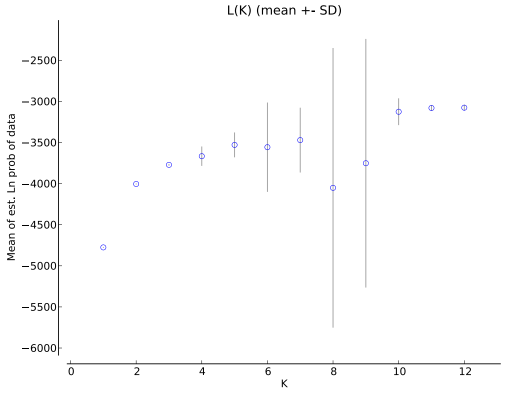

Including only unique MLGs

ΔK

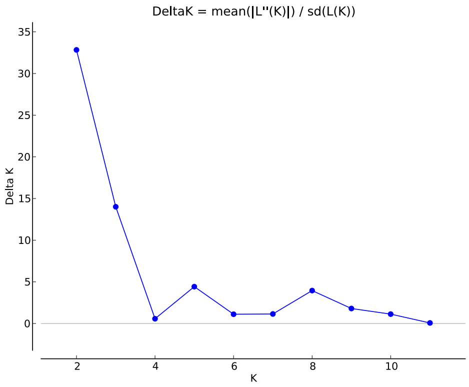

Maximum Likelihood

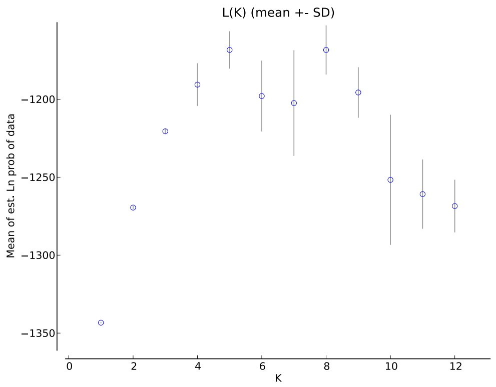

Supplementary material S9. Delta K (ΔK) and Maximum Likelihood plots from Structure analyses using two different databases: including all individuals or including only unique MLGs.

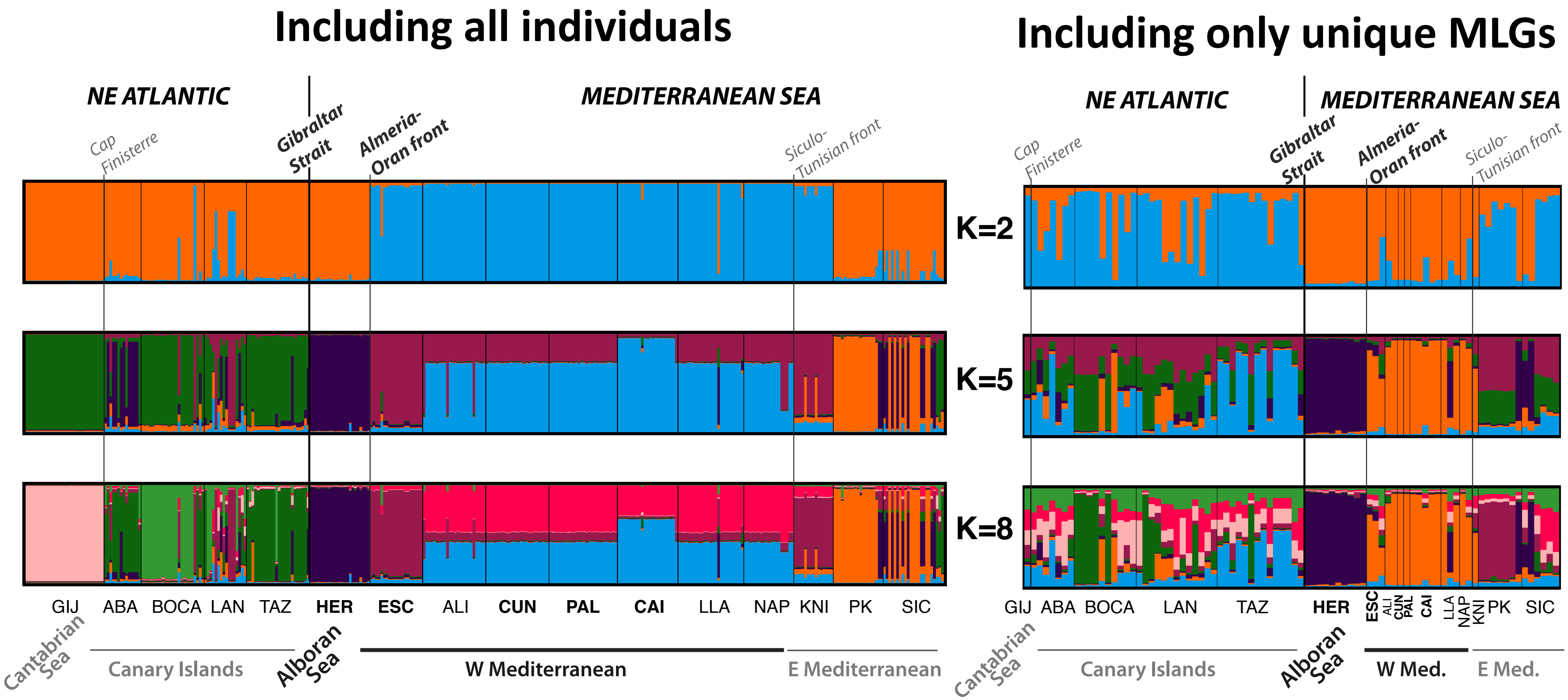

Supplementary material S10. Barplots from the Bayesian clustering analysis obtained in STRUCTURE for K=2, K=5, and K=8. On the left: results from the whole dataset, including all individuals. On the right: including only unique MLGs.
Major oceanographic areas and fronts are indicated.

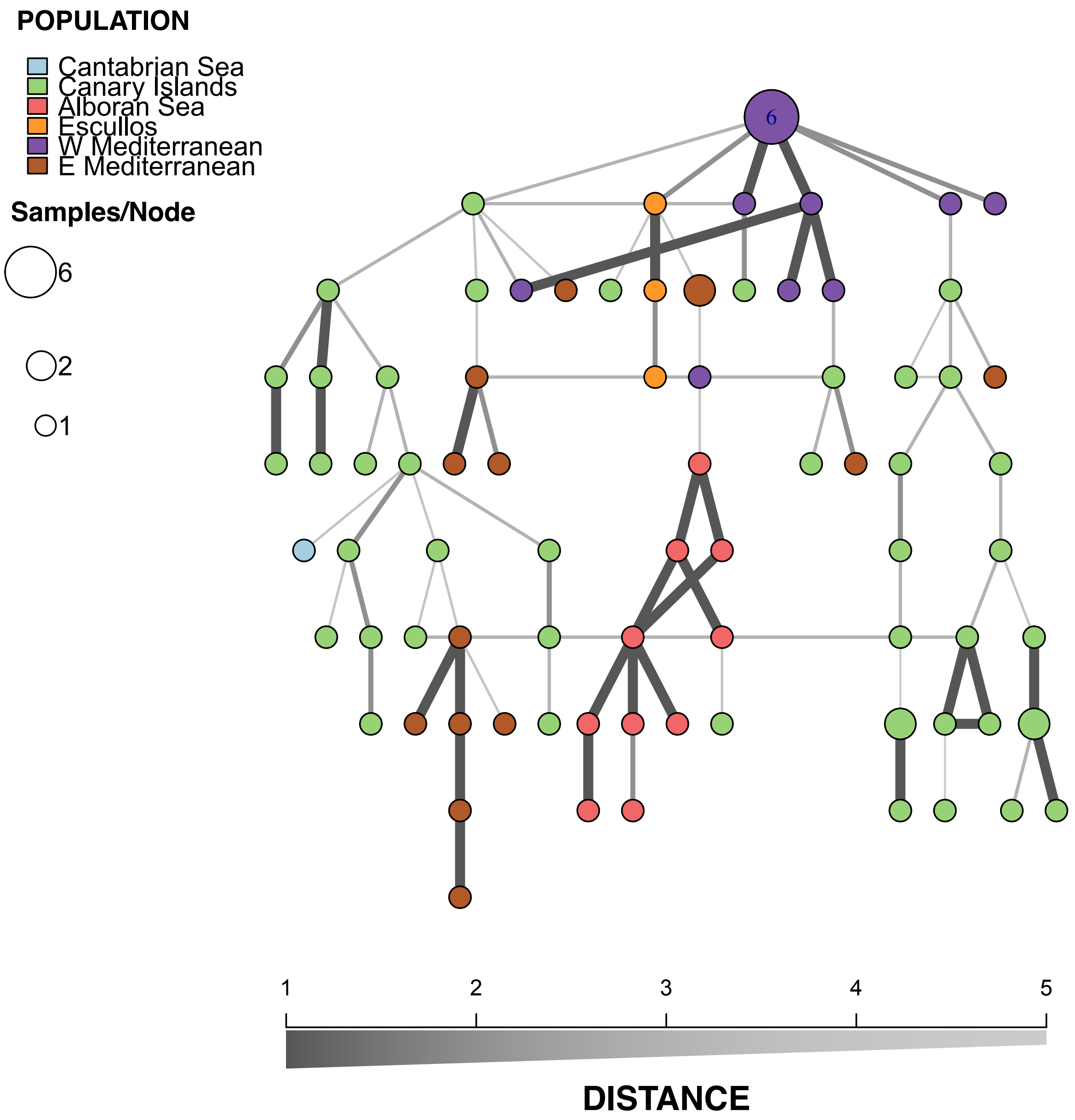

Supplementary material S11. Minimum spanning network from MLGs. Circles represent the different MLGs and connecting lines represent genetic distances measured by allele variants. Colours of the circles represent different geographical areas and colours and thickness of the connections are proportional to the genetic distances. Thick and dark connection lines represent only one allele-variant between MLGs.

Circles with a frequency (size) higher than one represent the same MLGs shared between populations. Therefore, the size of the circle represents the number of populations, within a geographical area, where that particular MLG has been detected.

The network including all individuals is not shown.

References:

1 Benjamini, Y. & Yekutieli, D. The control of the false discovery rate in multiple testing under dependency. *Annals of statistics*, 1165-1188 (2001).
